# Chemosensitivity to doxorubicin of ER-positive/HER2-negative breast cancers with high 21-gene recurrence score: A study based on *in vitro* chemoresponse assay

**DOI:** 10.1371/journal.pone.0187679

**Published:** 2017-11-08

**Authors:** Sung Gwe Ahn, Soong June Bae, Changik Yoon, Yoon Jin Cha, Hak Woo Lee, Seung Ah Lee, Joon Jeong

**Affiliations:** 1 Department of Surgery, Gangnam Severance Hospital, Yonsei University College of Medicine, Seoul, Korea; 2 Department of Pathology, Gangnam Severance Hospital, Yonsei University College of Medicine, Seoul, Korea; 3 Department of Surgery, CHA Bundang Medical Center, CHA University, Seongnam, Korea; University of Texas MD Anderson Cancer Center, UNITED STATES

## Abstract

**Aim:**

The 21-gene recurrence score (RS) predicts a clinical benefit of chemotherapy for individuals with ER-positive/HER2-negative breast cancer. Using *in vitro* chemoresponse assay, we compared the chemosensitivity according to RS in these patients.

**Method:**

Among the patients with Oncotype Dx assay, we identified 63 patients who had chemotherapy response assays to doxorubicin based on adenosine triphosphate. The degree of chemosensitivity to doxorubicin was translated into the cell death rate (CDR). The RS was also dichotomized with a cutoff of 26.

**Results:**

Of 63 patients, 34 (54%), 17 (27%), and 12 patients (19%) had a low, intermediate, and high RS, respectively. The mean CDR differed significantly according to categorized RS, with 17.3±10.8 in the low RS group vs. 23.6±16.3 in the intermediate RS group vs. 28.8±12.6 in the high RS group (*P* = 0.024, One-way ANOVA test). The mean CDR was significantly higher in the higher RS (26≥) group compared with the lower RS (<26) group (*P* = 0.025, the Student’s *t*-test), as well as in the high RS (>30) group compared with the low RS (<18) group (*P* = 0.012, the Student’s *t*-test). Also, continuous RS and CDR correlated positively (Pearson’s R = 0.337; P = 0.007). High RS demonstrated the odds ratio (OR = 26.33; 95% CI = 1.69–410.0) for predicting tumors with chemosensitivity on the multivariate analysis.

**Conclusions:**

The chemosensitivity measured by *in vitro* chemoresponse assay was different according to the RS. Our findings support that tumors with high RS has the chemosensitivity even though they are luminal/HER2-negative tumors.

## Background

It has been widely recognized that a substantial number of patients with estrogen receptor (ER)-positive breast cancer would have been adequately treated with endocrine therapy alone [1, 2]. The Early Breast Cancer Trialists meta-analysis have shown that the addition of chemotherapy does not offer significant benefit but exposes toxicity from cytotoxic chemotherapy in a majority of patients with ER-positive/human epidermal growth factor receptor2 (HER2)-negative tumors [3, 4]. To select patients who could be spared adjuvant chemotherapy, the use of molecular signatures and their validations are regarded as a high priority [5].

Currently, several multigene assays are available for clinicians to identify the appropriate patients who might derive a clinical benefit from chemotherapy [6]. Among several multigene assays which help to determine chemotherapy or not in ER-positive tumors, the 21-gene recurrence score (RS) became the first clinically validated multi-gene assay with the use of archival tumor specimens from completed studies that used a prospective–retrospective design [7, 8]. Also, it has been incorporated into clinical guidelines concerning treatment decisions with a high level of recommendation^6^ and has become widespread in actual practice [9].

It is easily elicited that survival benefit by the addition of adjuvant chemotherapy is associated with responsiveness to chemotherapy. Thus, it might be assumed that tumors with high RS would have a relatively higher response to neoadjuvant chemotherapy (NAC) compared with tumors with low or intermediate RS. Indeed, previous studies have shown that tumors with high RS have a higher rate of pathologic complete response (pCR) in neoadjuvat setting [10, 11].

To predict responsiveness to chemotherapy in individual tumors using *in vitro* assays, several methods are developed and tested. Among these assays, in vitro CRAs using adenosine triphosphate (ATP-CRAs) can be performed relatively quickly and have overcome the technical problems caused by fibroblast contamination [12, 13]. Previously we provided evidence that ATP-CRAs for breast tumors could effectively reflect the tumor response to chemotherapy observed in neoadjuvant setting [12].

In this study, we wondered whether tumors with high RS might have a higher chemosensitivity to doxorubicin superior to tumors with low RS based on ATP-CRAs. Using *in vitro* assay, we aim to know biological difference among ER-positive/HER2-negative tumors according to RS in context of chemotherapy-responsiveness.

## Methods

### Patients

The institutional review board of Gangnam Severance Hospital, Yonsei University, Seoul, Korea, approved the study to be in accordance with good clinical practice guidelines and the Declaration of Helsinki. The need for informed consent was waived due to the retrospective design under the approval of the institutional review board. Between August 2011 and June 2016, 244 patients underwent Oncotype DX testing at Gangnam Severance Hospital and Severance Hospital, Yonsei University College of Medicine. All patients had ER-positive, HER2-negative breast cancer. Of these patients, we identified 63 patients who donated their tumor samples for ATP-CRAs with doxorubicin. The degree of chemosensitivity to doxorubicin was translated into the cell death rate (CDR). Raw data of these patients is provided online (S1 Data).

### Immunohistochemical method

For our IHC study of four markers, formalin-fixed, paraffin-embedded tissue sections obtained from surgical specimens were stained with appropriate antibodies specific for the ER (1:100 clone 6F11; Novocastra, Newcastle upon Tyne, UK), progesterone receptor (PR; clone 16; Novocastra), and Ki-67 (MIB-1; Dako, Glostrup, Denmark). ER and PR IHC test results were stratified into four groups using the modified Allred system: strong, Allred score 7–8; moderate, Allred score 5–6; weak, Allred score 2–4; and negative, Allred score 0–1 [14]. Ki67 expression was measured by an experienced pathologist and presented as a percentage score (range 0–100%) of positive tumor cells.

### Oncotype Dx assays

RS is calculated by the Oncotype Dx assay. It is a continuous score that is classified into the following categories: low risk (RS < 18), intermediate risk (RS 18–30), and high risk (RS≥31). The Oncotype DX assay was performed using RNA extracted from formalin-fixed paraffin-embedded tissue and supplied by Genomic Health (Redwood City, CA, USA). After a review of hematoxylin and eosin-stained slides to determine whether sufficient invasive breast cancer was present and whether manual microdissection was indicated, RNA was extracted from the unstained sections. Cases with no cancer (depleted by prior tissue studies) or with cancer cells occupying <5% of the section area were excluded from the assay. The RS was also dichotomized with a cutoff of 26 following an ongoing clinical trial as TAILORX.^2,14^

### *In vitro* chemoresponse assays using adenosine triphosphate (ATP-CRAs)

Tumor tissues were stored in Hank’s balanced salt solution (Gibco BRL, Rockville, MD, USA) containing100 IU/ml penicillin (Sigma, St. Louis, MO, USA), 100 mg/ml streptomycin (Sigma), 100 mg/ml gentamicin (Gibco BRL), 2.5 mg/ml amphotericin B (Gibco BRL) and 5% fetal bovine serum (Gibco BRL) on operation day. After histological evaluation, within 24 h after operation, the tumor tissues were incubated in a mixture of dispase (Sigma), pronase (Sigma) and DNase (Sigma) for 12–16 hours at 37°C. Isolated cells were separated from tissue fragments by passing through a cell strainer (BD Falcon, Bedford, MA, USA). Tumor cells were separated from dead cells and red blood cells using Ficoll gradient (1.077 g/ml) centrifugation at 400g for 15 min. When a sufficient amount of cells were isolated, blood-derived normal cells were removed using CD45 antibody-conjugated magnetic beads (Miltenyi Biotech, Auburn, CA, USA). The separated tumor cell preparation was suspended in IMDM (Gibco BRL) including 10% FBS and antibiotics, as mentioned above. Cells were diluted to concentrations between 5000 and 20,000 viable cells/100 ml for plating into a 96-well ultralow attachment microplate (Costar, Cambridge, MA, USA), with or without anti-cancer drugs, and cultured for 48 h in a CO2 incubator.

Treated drug concentrations (TDC) were determined by preliminary experiments that showed the scattered distribution of cell death from each specimen The TDC for doxorubicin was 1.5 mg/ml; To measure ATP levels, ATP in the cell lysate was reacted with luciferin (Roche, Mannheim, Germany) and excessive luciferase was measured using a Victor 3 multi-label counter (PerkinElmer, Boston, MA, USA). Excel-based raw data were analyzed using Report Maker version 1.1 (ISU ABXIS, Seoul, Korea). Briefly, the cell death rate for each drug was calculated as follows: **Cell death rate (CDR) (%) = (1 - [mean luminescence in treated group/mean luminescence in untreated controls group])X100**. To calculate the intra-assay mean coefficient of variation (CV), luminescence values of each specimen were measured 3–6 times in negative and positive control groups. We next determined whether the measured values at 280 pg ATP were higher than at 105 pg ATP. The test was considered a failure if microorganism contamination was present, there was an inadequate number of cells, or if the intra-assay mean CV exceeded 30. In addition, if the measured values in the untreated control group were lower than that in the positive group (105 pg ATP), the specimen was considered to have unacceptable viability.

### Statistical analysis

Discrete variables were compared using the χ2 test or Fisher’s exact test. Student’s t-test or a one-way analysis of variation (ANOVA) test was used to compare means. Pearson’s R was calculated to measure the correlative value between the RS and CDR. The binary logistic regression models were employed to predict tumors with chemosensitivity. SPSS version 18 (SPSS Inc., Chicago, IL, USA) was used to perform the statistical analyses. Statistical significance was defined as a P-value <0.05.

## Results

### Baseline characteristics

The baseline patient characteristics are presented in Table 1. Sixty-three patients with ER-positive, HER2-negative tumors were included in the analyses. The median age of these patients was 52 years (range: 28–75 years). Eleven patients had node-positive disease, and 3 had micrometastases. No patient in the study population had a stage higher than IIB.

**Table 1:** Baseline characteristics.

Variables	Lower RS (N = 48)	Higher RS (N = 15)	*P*-value
**Age (median, range)**	49 (28–75)	56 (38–62)	0.344
**Tumor size (median, range)**	1.8 (0.9–4.0)	2.2 (0.9–3.5)	0.210
**T stage**			0.073
T1	30 (62)	5 (33)	
T2	18 (38)	10 (67)	
**N stage**			0.244
0	35 (73)	14 (93)	
Micrometastasis	2 (4)	1 (7)	
N1	11 (23)	0 (0)	
**Stage**			0.232
I	23 (48)	4 (27)	
II	25 (52)	11 (73)	
**Grade**			0.142
I or II	40 (85)	10 (67)	
III	7 (15)	5 (33)	
**Estrogen receptor** ^**a**^			1.000
High	39 (81)	12 (80)	
Low	9 (19)	3 (20)	
**Progesterone receptor** ^**a**^			0.002
High	33 (69)	3 (20)	
Low	15 (31)	12 (80)	
**Ki67**			<0.001
≥20	7 (15)	10 (67)	
<20	41 (85)	5 (33)	

^a^ High, Allred score 5–8; Low, Allred score 0–4

Thirty-four (54%), 17 (27%), and 12 patients (19%) had a low, intermediate, and high RS, respectively. Furthermore, 15 patients (24%) had tumors with higher RS (≥26), whereas 48 (76%) had tumors with lower RS (<26). The lower RS group tended to have tumors with higher PR expression and lower Ki67 expression, compared with the higher RS group.

### Comparisons cell death rates according to categorized RS

**Fig 1:**
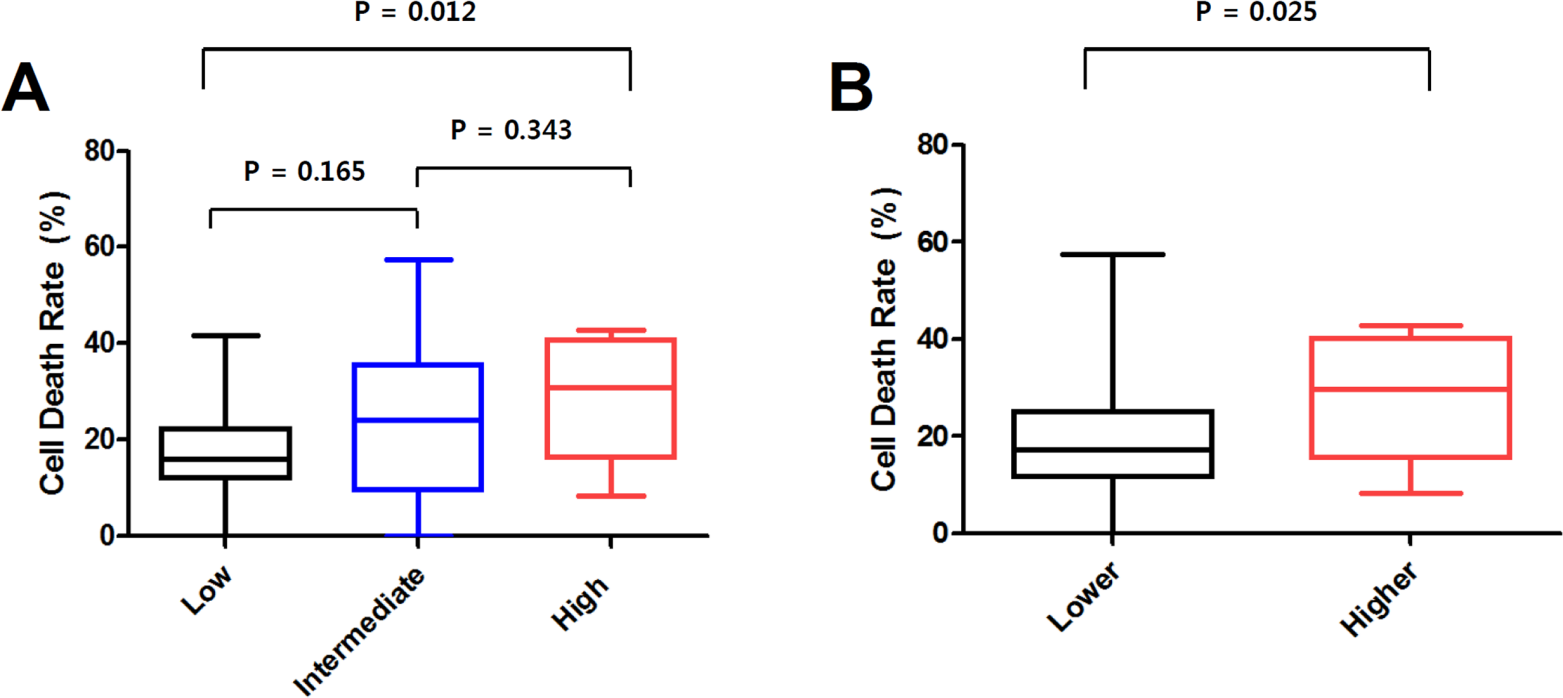
Distributions and means of cell death rate (CDR) according to categorized recurrence score (RS). (A) The mean CDRs differed significantly among the three groups (*P* = 0.024, One-way ANOVA test). The mean CDR was significantly higher in the high RS group compared with the low RS group (*P* = 0.012, the Student’s *t*-test). However, a significant difference of CDR between the low and intermediate RS groups or the intermediate and high RS groups was not observed. (B) The mean CDR was significantly higher in the higher RS group compared with the lower RS group (*P* = 0.025, the Student’s *t*-test).

The mean CDR differed significantly according to categorized RS, with 17.3±10.8 in the low RS group vs. 23.6±16.3 in the intermediate RS group vs. 28.8±12.6 in the high RS group (*P* = 0.024, One-way ANOVA test; Fig 1A). The mean CDR was significantly higher in the high RS group compared with the low RS group (*P* = 0.012, the Student’s *t*-test). However, a significant difference of CDR between the low and intermediate RS groups or the intermediate and high RS groups was not observed.

Moreover, when we compared the mean CDR according to dichotomized RS, a significant difference was observed, with 19.1±13.2 in the lower RS group vs. 27.9±12.5 in the higher RS group (*P* = 0.025; Fig 1B).

### Correlation between continuous RS and continuous CDR

**Fig 2:**
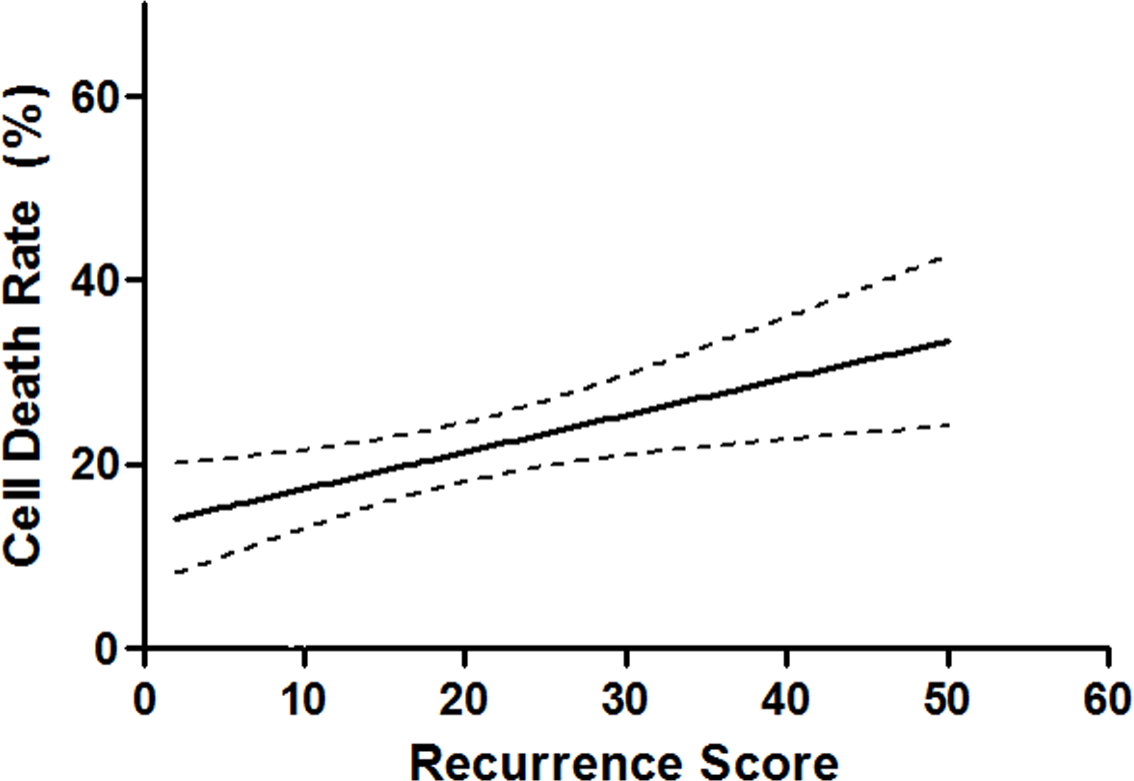
Cubic spline curve of the cell death rate to doxorubicin as a function of recurrence score. A significant positive correlation was observed between the two continuous parameters (Pearson’s R = 0.337; *P* = 0.007).

Pearson's R test was performed to explore the relationship between continuous RS and continuous CDR. A significant positive correlation was observed between the two continuous parameters (Pearson’s R = 0.337; P = 0.007; Fig 2).

### Logistic regression analysis

**Fig 3:**
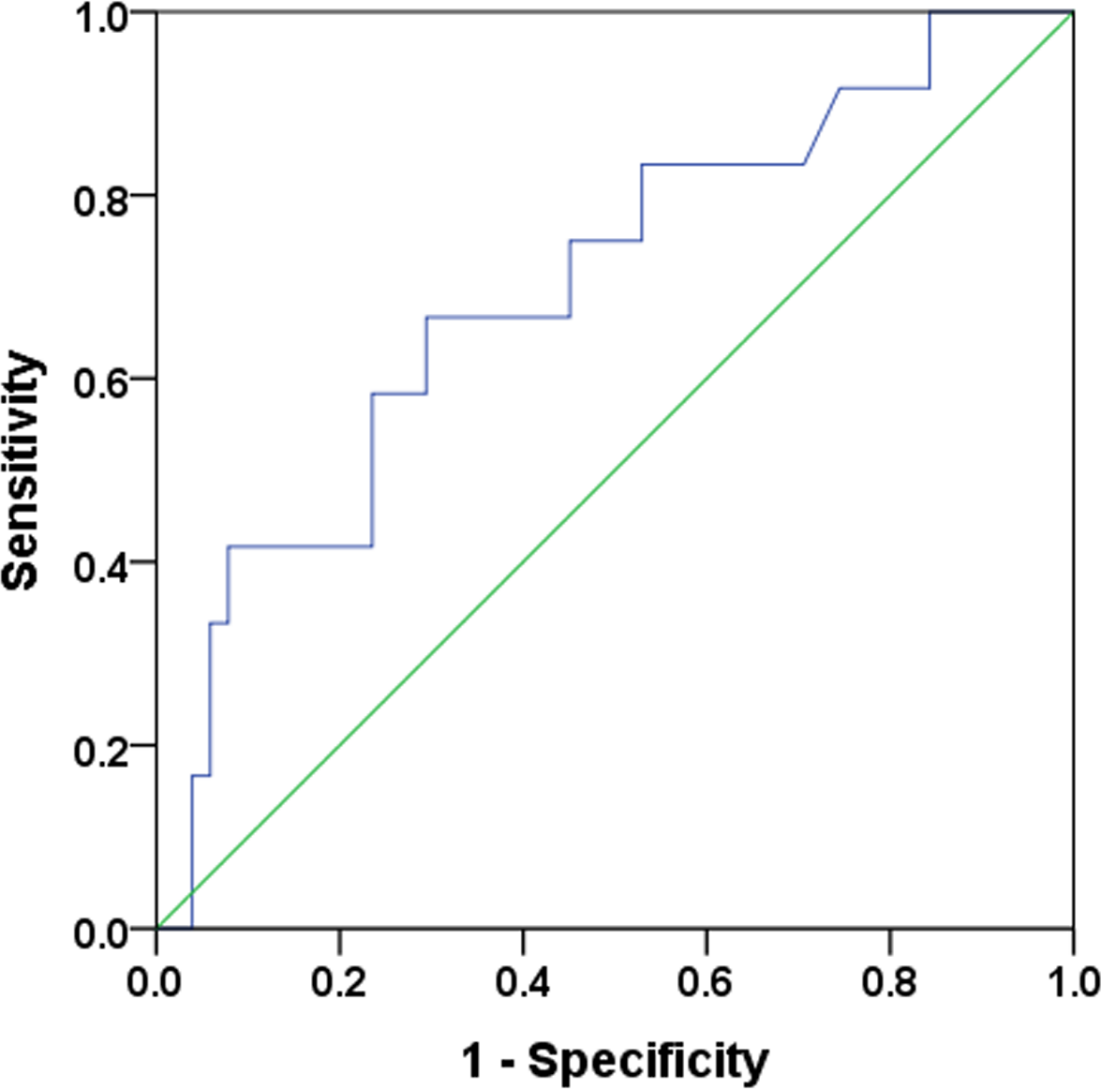
The receiver operating characteristic (ROC) curve for cell death rate (CDR) in relation to high RS. The ROC curve yielded an area under the curve of 0.701 (95% CI, 0.533–0.869, *P* = 0.031). Youden’s index was the highest for CDR of 28.

The area under the receiver operating characteristic curve for continuous CDR was 0.701 (95% CI, 0.533–0.869, *P* = 0.031) for distinguishing high RS from low or intermediate RS (Fig 3). Youden’s index was the highest for CDR of 28.1. As a result, we defined the CDR cutoff as 30 to identify tumors with chemosensitivity within this study. Multivariate analysis with biologic parameters revealed that RS, ER and PR expression remained independent variables associated with chemosensitivity (Table 2). High RS demonstrated the odds ratio (OR = 26.33; 95% CI = 1.69–410.0) for predicting tumors with chemosensitivity on the multivariate analysis.

**Table 2:** Binary logistic regression analysis for factors associated with chemosensitivity (cell death rate≥30%).

Variables	*P* value	Odds Ratio	95% CI
**Recurrence score**			
Low	Ref		
Intermediate	0.057	4.42	0.96–20.44
High	0.020	26.33	1.69–410.0
**Histologic grade**			
I or II	Ref		
III	0.361	2.11	0.43–10.48
**Estrogen receptor** ^**a**^			
Higher	Ref		
Lower	0.020	26.6	1.69–418.64
**Progesterone receptor** ^**a**^			
Higher vs. Lower	Ref		
Lower	0.037	16.15	1.18–221.94
**Ki67**			
<20%	Ref		
≥20%	0.646	1.49	0.27–8.27

^a^ Higher, Allred score 5–8; Lower, Allred score 0–4.

## Discussions

Using *in vitro* chemoresponse assay, we provided evidence that tumors with high or higher RS have superior chemo-responsiveness compared with tumors with low or lower RS. A linear correlation between continuous RS and continuous CDR was also observed. Finally, we showed that high RS was associated with chemosensitivity to doxorubicin independent of other biologic parameters in ER-positive/HER2 negative tumors.

Our findings are in the line with previous studies that tumors with high RS have a higher rate of pCR in neoadjuvant setting. In the study by Gianni et al, they showed that RS was positively associated with the likelihood of pCR [10]. Another study by Yardley et al. also supported this finding that achievement of pCR was only observed in tumors with high RS (0 of 36 with low or intermediate RS vs. 19 of 72 with high RS) [11]. Although these studies also included ER-negative tumors, they provided evidence that tumors with high RS had a higher probability of pCR compared with tumors with low or intermediate RS in neoadjuvant setting. Conversely, the recent study including ER-positive/HER2-negative tumors alone showed that clinical response rate or tumor size reduction rate did not correlate with continuous or categorized RS [15]. Notably, there is no case with pCR in the study. The rarity of pCR might be associated with a discrepancy among those studies.

Also, there is another study which delivers NAC in ER-positive/HER2-negative patients with higher RS (≥26) using core biopsy samples [16]. In these, the rate of pCR in both breast and axilla was 14.3% (2 of 14). Despite of small number of patients, it supports that tumors with higher RS have chance of pCR even though they are luminal/HER2-negative tumors.

A less sensitivity to chemotherapy in low RS tumors observed in our *in vitro* data also supports that tumors with low RS would be marginally benefited by the addition of chemotherapy. A recent report from the subgroup of the TAILORX trial also shows that endocrine alone is adequate treatment for those with tumors that had a low RS among patients with hormone receptor–positive, HER2-negative, axillary node–negative breast cancer [17]. In the study, they had very low rates of recurrence at 5 years with endocrine therapy alone.

A small number of patients is major limitation in our study. The absence of clinical outcome in relation to ATP-CRA and the restriction of the clinical application of ATP-CRA guided chemotherapy is another weakness of our study. Although this study was conducted using a retrospective design and had several limitations, our study provide *in vitro* data supporting that tumors with high RS have an increased responsiveness to chemotherapy.

In conclusions, the chemosensitivity to doxorubicin measured by in vitro chemoresponse assay was different according to the categorized RS. Our findings support that tumors with higher RS has the higher chemosensitivity even though they are luminal/HER2-negative tumors.

## Supporting information

S1 Data Raw data of patients is provided online. (XLSX)
